# Comparison of the efficacy of acupuncture at the TUNG’s extra points and the traditional Chinese medicine points for elderly patients with chronic low back pain in Thailand

**DOI:** 10.1007/s11726-022-1331-7

**Published:** 2022-11-04

**Authors:** Poonyaphat Siriteerathitikul, Saengchai Wongmanakul, Monticha Kunyalue, Punyawee Khamthai

**Affiliations:** Department of Traditional Chinese Medicine, School of Public Health, University of Phayao, Phayao, 56000 Thailand

**Keywords:** Acupuncture Therapy, TUNG’s Extra Points, Acupuncture Points, Pain Measurement, Low Back Pain, Aged, 针刺疗法, 董氏奇穴, 针刺穴位, 疼痛测评, 腰痛, 老年人, R246.2, A

## Abstract

**Objective:**

To compare the efficacy of acupuncture at TUNG’s extra points and traditional Chinese medicine (TCM) points for elderly patients with chronic low back pain (CLBP) in Thailand.

**Methods:**

A single-blinded, randomized controlled trial with 84 elderly volunteers with CLBP was designed. The patients were randomly assigned either to the group getting acupuncture at TUNG’s extra points or to the group getting acupuncture at TCM points. The treatment period was 30 min per session for seven consecutive days. Before and after treatment, the score of the numeric rating scale (NRS), the back range of motion (BROM), and the back strength were measured and compared.

**Results:**

After treatment, both groups were found with decreased NRS scores and increased BROM (*P*<0.05), but with no statistical difference in their back strength in comparison with that before treatment in the same group (*P*>0.05). Regarding the between-group comparisons, no significant differences were found in the NRS score or BROM in the direction of forward flexion and right lateral flexion or the back strength after treatment (*P*>0.05). However, statistical differences were found in the BROM in directions of back extension (*P*<0.01) and left lateral flexion (*P*<0.05).

**Conclusion:**

Acupuncture at TUNG’s extra points can decrease the low back pain NRS score and increase the back strength and the BROM in directions of forward flexion and right lateral flexion, equivalent to acupuncture at TCM points. Acupuncture at TCM points has a better effect in increasing the BROM in directions of back extension and left lateral flexion; acupuncture at TUNG’s extra points is suitable for elderly CLBP patients, and it should be supported and promoted.

## Abstract (zh)

**目的:**

比较针刺董氏奇穴和中医传统穴位治疗泰国老年慢性腰痛(CLBP)患者的疗效。

**方法:**

采用单盲、随机 对照试验设计, 纳入84 名慢性腰痛老年患者。所有患者被随机分为两组, 一组接受董氏奇穴针刺治疗, 另一组接受 传统中医(TCM)穴位针刺治疗。每次治疗30 min, 连续治疗7 d。治疗前后进行数字评定量表(NRS)评分、背部活动度 (BROM)及背部力量的测量和比较。

**结果:**

与同组治疗前比较, 治疗后两组NRS评分降低, BROM增加(*P*<0.05), 但腰部 力量无统计学差异。组间比较, 治疗后两组NRS评分、前屈及右侧屈BROM、腰部力量方面的差异均无统计学意义 (*P*>0.05), 而两组BROM在后伸(*P*<0.01)和左侧屈(*P*<0.05)方面的差异有统计学意义。

**结论:**

针刺董氏奇穴在降低腰部疼 痛NRS评分, 增加腰部力量, 以及增加腰前屈及右侧屈活动度方面与针刺TCM穴位的效果相当. 针刺传统穴位在增加 腰后伸及左侧屈活动度方面效果更佳; 针刺董氏奇穴适合老年CLBP患者, 应得到支持推广。

**【中图分类号】**R246.2　　**【文献标志码】**A

Chronic low back pain (CLBP) is an increasingly common pain experienced between the 12th rib and coccyx area[1], with 4.2%-25.4% of the world population having it[2-3]. The *Global Burden of Disease Study* reports that low back pain is the top cause of disabilities found in elderly patients aged 80 years and above[4-5]. Although older adults' low back pain is not severe on the numeric rating scale (NRS) or acute, chronic pain is prevalent, which likely leads to disability[6]. At present, the elderly population has increased all over the world, and Thailand has a good proportion of aged people, which is 20%, higher than that of the newborn population at a ratio of 1:5 to the

**Author**: Poonyaphat SIRITEERATHITIKUL, M.M., lecture.
E-mail: poonyaphat.si@up.ac.th

| © The author(s) for open access article 2023
Springer whole Thai population[7]. Therefore, elderly people are the majority of the current Thai society, so their health is crucial for social happiness. According to the report on Thai people's health, the highest percentage of disabilities found in the northern region of Thailand is higher compared with the elderly population in other regions. The common elderly health problem is low back pain due to physical deterioration leading to lower functional effectiveness in the older ages[8]. In addition, another cause is improper postures from daily activities, which result in intervertebral disc compressive forces. They may also have a history of misusing their back by bowing down to lift heavy things, moving in wrong postures, or standing or sitting at a stretch for a long time[9]. Getting injuries in the spinal muscles and ligaments is also a contributing factor. These causes affect low back muscle pain and fatigue, leading to limitations in daily activities and work, ultimately causing disability and limited physical abilities[10-11]. They may interfere with neuromuscular systems as they cause abnormal physical functions in walking, running, working, or doing various activities, wasting one's time and money[12]. Particularly, older people in rural areas usually lack access to good diagnoses, treatments, and various facilities compared to those living in urban areas[7-8].

There are several CLBP treatments, such as non-steroidal anti-inflammatory drugs (NSAIDs) and muscle relaxants as well as non-drug treatments such as bed rest, massage, physical therapy, and Chinese medicine of acupuncture[13]. In the current CLBP treatment, acupuncture is more commonly used due to its high effectiveness and safety[14]. Acupuncture is a part of traditional Chinese medicine (TCM) that punctures the needles at the points in the flowing meridian area. The selected points are the traditional acupuncture points and the tender points[15]. The selected points may be increased or decreased depending on the symptoms. In addition, one acupuncture type is Master TUNG's acupuncture, invented by TUNG Ching Chang, which uses a small number of needles to puncture the reflexology points far from the pain location while applying pressure through muscle or joint motions at the painful area[16]. The research team thinks that acupuncture technique at the TUNG's extra points can be done by using only three needles as it will be more convenient for older people in rural areas. However, this technique is rarely studied, so it has limited empirical findings to support the said effectiveness. Therefore, this comparative study was designed as a single-blinded, randomized controlled trial in which the raters were unaware of the treatment method used on the study volunteers or the other researchers who performed the acupuncture treatment. The purpose of the study was to compare the effectiveness of acupuncture at the TUNG's extra points and acupuncture at the TCM points in the pain NRS score, back range of motion (BROM), and low back strength of the elderly patients with CLBP in the rural area in Mae Ka Community, Muang District, Phayao Province in the northern region of Thailand. That is to confirm the treatment results of acupuncture at the TUNG's extra points and to compare the results to analyze which acupuncture method could better reduce the low back pain and increase the BROM and low back strength. The research hypothesizes that the effectiveness of acupuncture at the TUNG's extra points should be equivalent to its effectiveness at the TCM points.

## 1 Clinical Materials

### 1.1 Diagnostic criteria

This study referred to the diagnostic criteria for CLBP in the related literature[17]. Recurrent CLBP in the past three months; assessment of prognostic factors such as work-related factors, psychosocial distress, depressive mood, the severity of pain and functional impact, prior episodes of low back pain, extreme symptom reporting, and patient's expectations; imaging was not recommended unless a specific cause was strongly suspected; magnetic resonance imaging was the best option for radicular symptoms, discitis, or neoplasm; plain radiography was the best option for structural deformities[18].

### 1.2 Inclusion criteria

The inclusion criteria included male or female volunteers at the age of 60 years and above with primary modern medical diagnoses showing CLBP with an average NRS score of 5-8 points for a period of more than three months. The volunteers were also selected from those who did not suffer from pain in the legs and had never received any acupuncture treatment before. The volunteers were requested not to do activities with risk factors that would cause more pain and not receive any other treatment during the study period. If any volunteers were not compliant with these inclusion criteria, they would be withdrawn from the study. The volunteers with eligible qualifications and willingness to join this study were informed in detail of the study procedures, and they signed their written consent.

### 1.3 Exclusion criteria

The exclusion criteria were also applicable for low back pain caused by problems related to herniated disc or degenerative disc, blood vessels, spinal cord injuries caused by accidents, history of back and hip surgery, infection and severe diseases. Those who have received treatment from other methods at least one month before the study period were also excluded.

### 1.4 Elimination and dropout criteria

Those who had adverse events or specific physiological changes were not suitable for continuing the trial; those who dropped out halfway; patients with incomplete data that would affect the accuracy of observation and statistics.

### 1.5 Statistical methods

All data from this study were analyzed with the SPSS version 26.0 software. The volunteers' general data were analyzed in the descriptive statistics by distributing them according to frequency, percentage, means, and standard deviation. The comparative data within the group regarding the NRS score, BROM, and back strength before and after the trial were analyzed with the dependent *t*-test, whereas the comparative data between groups were analyzed with the independent *t*-test at a statistical significance of $P<0.05$.

### 1.6 General data

The data were then collected at Mae Ka Health Promoting Hospital, Muang District, Phayao Province in Thailand. The population and samples of the study included 84 elderly patients with CLBP aged 60 years and above. The study period was between August 2021 and February 2022.

The simple random sampling with the lottery without replacement method was used to assign the 84 volunteers to each group randomly: a group getting acupuncture at the TUNG's extra points or a group getting acupuncture at the TCM points. In this study, there were 150 volunteers, but 66 not compliant with the inclusion criteria and thus screened out from the study. Therefore, the remaining 84 volunteers in the study were randomly assigned to one of the groups: the group that received acupuncture at the TUNG's extra points or the group that received acupuncture at the TCM points, with 42 volunteers in each group.

During the study, it was found that in the group getting acupuncture at the TUNG's extra points, 11 volunteers were absent from the appointments, and three volunteers did not conform to the conditions. Therefore, 28 volunteers got the complete treatment and were assessed on all variables before and after the trial. In the group getting acupuncture at the TCM points, 10 volunteers were absent from the appointments, and two did not conform to the conditions. Thus, 30 volunteers received the complete treatment and were assessed on all variables before and after the trial (Figure 1).

The general data of the volunteers in the two groups were not significantly different with regard to their gender, age, body mass index, and the period of getting CLBP ($P>0.05$). This indicated that the demographic data of the two groups were similar (Table 1).

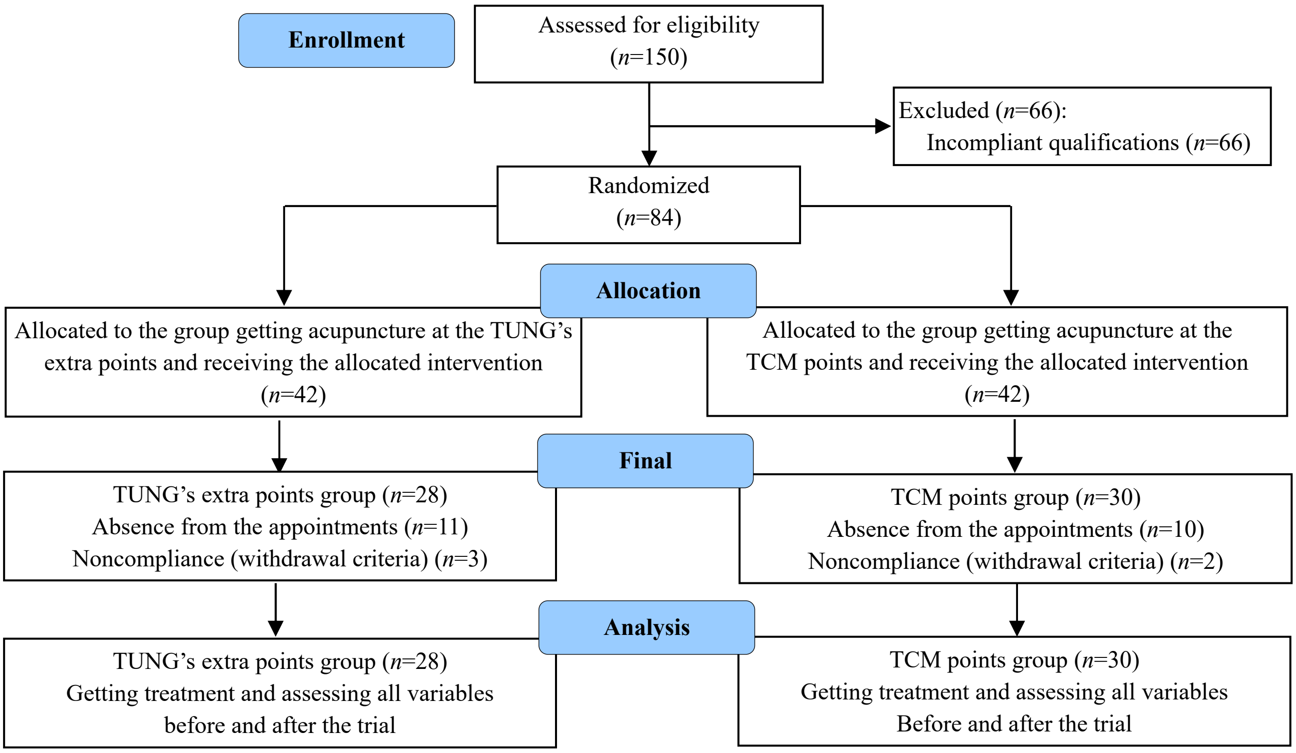

Note: TCM=Traditional Chinese medicine.

**Figure 1 Flow chart of the study according to the Consolidated Standards of Reporting Trials (CONSORT)[19]**

**Table 1 Comparison of the general data between the two groups**

| Group | $n$ | Gender/case | | Age/year ($\bar{x}\pm s$) | Body mass index/(kg·m$^{-2}$) ($\bar{x}\pm s$) | Pain period/year ($\bar{x}\pm s$) |
|---|---|---|---|---|---|---|
| | | Male | Female | | | |
| TUNG's extra points | 28 | 9 | 19 | 64.2±13.8 | 23.00±3.95 | 7.75±2.38 |
| TCM points | 30 | 7 | 23 | 63.0±11.4 | 23.39±4.09 | 7.27±2.50 |
| Statistical value | | 0.563$^{1)}$ | | 0.346$^{2)}$ | -0.363$^{2)}$ | -0.536$^{2)}$ |
| $P$-value | | 0.453 | | 0.730 | 0.718 | 0.594 |

Note: TCM=Traditional Chinese medicine; 1) $\chi^2$-value; 2) $t$-value.

## 2 Treatment Methods

The acupuncture treatments were performed by other researchers. They started by disinfecting all the points with 70% alcohol, which is prescribed in the National Certification Commission for Acupuncture and Oriental Medicine (NCCAOM) standard[20].

### 2.1 TCM points group

For the TCM points group, acupuncture was performed at the TCM points according to the TCM therapy.

Points: Ashi points (trigger points), lumbar ($L_1$-$L_5$) Jiaji (EX-B2) points, Dachangshu (BL25), Shenshu (BL23), Yaoyan (EX-B7), and Weizhong (BL40), (Figure 2)[21].

The acupuncture was performed while the patients were in a prone position. Then, the needles of 0.25 mm in diameter and 40 mm in length were inserted into the selected points with 1.5-2.5 cm in depth, depending on the thickness of the volunteers' skin and muscles. After that, the needles were performed until the volunteers felt stiffness or tightness (Deqi) at all points. The needles were withdrawn after being retained for 30 min. The treatment was performed in seven sessions that spanned seven consecutive days.

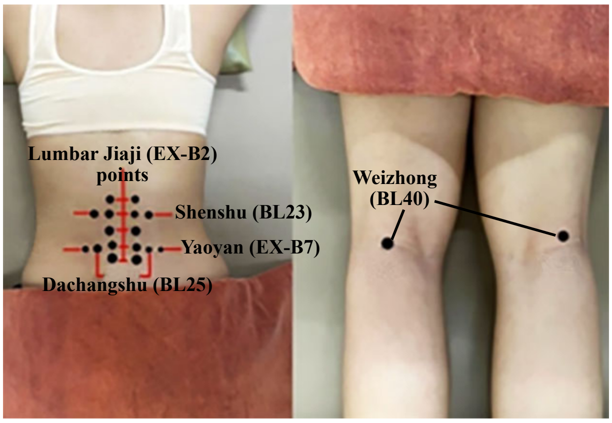

**Figure 2 Traditional Chinese medicine points**

### 2.2 TUNG's extra points group

For the TUNG's extra points group, the acupuncture was performed at the TUNG's extra points according to TUNG's acupuncture at Linggu (22.05) and Dabai (22.04) points. Linggu (22.05) is on the dorsum of the hand, at the junction of the first and second metacarpal bones, on the Large Intestine Meridian, also opposite and articulate Chongxian (22.02) on the palmar surface. Dabai (22.04) is on the dorsum of the hand, in a depression 1 Cun distal to the junction of the first and second metacarpal bones; it is on the Large Intestine Meridian, distal to Hegu (LI4), or overlapping with Sanjian (LI3)[22]. The Dao Ma Zhen acupuncture method was used after Linggu (22.05) and Dabai (22.04) points were punctured, and another point was punctured in the middle of the two points on the same plane containing the three points on the hand (Figure 3). The researchers considered acupuncture on the left or right hand depending on the location of the pain. For left low back pain, acupuncture was applied to the right hand. For right low back pain, the left hand was treated. And if the pain was all over the back, acupuncture would be applied to both hands[23].

Researchers used needles of 0.25 mm in diameter and 25 mm in length to puncture by 1.0-1.5 cm depth. Next, the needles at the three points were stimulated until the volunteers felt stiff or tight (Deqi), during which the volunteers were requested to move the low back painful area gradually. Then, the needles were left there for 5 min and stimulated manually at the 3rd and 5th minutes. The treatment was performed in seven sessions that spanned seven consecutive days.

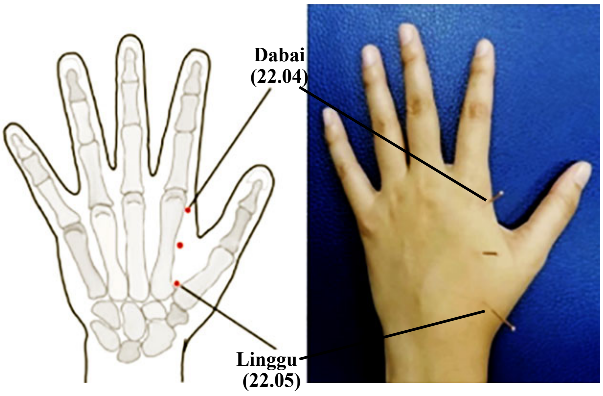

**Figure 3 TUNG's extra points**

## 3 Results Observation

### 3.1 Observation items

3.1.1 NRS score[24]

All volunteers were requested to rate their pain on the NRS that was in a range of 0-10 points. The score was interpreted according to the severity level: mild pain (1-3 points), moderate pain (4-6 points), and severe pain (7-10 points). These results were recorded before and after the treatment program.

3.1.2 BROM

A dual inclinometer was used to test the BROM in flexion and extension directions[25]. The bubble inclinometer was used to test the BROM in the directions of left lateral flexion and right lateral flexion[26]. All volunteers were tested on their BROM in four directions. The dual inclinometer was placed at $S_2$ and $T_1$ for testing back flexion and extension. Then the bubble inclinometer was placed at $T_{12}$ for testing back left lateral flexion and back right lateral flexion. Each direction was tested 3 times, and the average of these three results was recorded before and after the treatment program.

3.1.3 Back strength

The version of Takei T.K.K. 5402 (Japan) was used for measuring back strength[27]. All volunteers were measured on their back strength by using the back and leg dynamometer. Each volunteer was requested to stand upright on the base of the dynamometer with their feet shoulder-width apart. They should let their arms hang straight down to hold the center of the bar with both hands, with their palms facing toward the body. The participant was also asked to adjust the chain so that the knees would be stretched straightly at 180°. Then they were asked to pull as hard as possible on the chain, keeping their arms straight. The participants were asked to pull against the weight steadily, keeping their feet flat on the base of the dynamometer. Each direction was tested twice, and the best results were recorded before and after the treatment program[28].

### 3.2 Criteria for clinical efficacy

Efficacy was evaluated based on the change in the NRS score before and after treatment.

Markedly effective: The NRS score dropped by two or more levels.

Effective: The NRS score dropped by one level.

Invalid: The NRS score did not show significant changes.

### 3.3 Results

3.3.1 Comparison of the total effective rate

After treatment, the total effective rate of the TUNG's extra points group was 96.4% and 96.7% in the TCM points group, indicating that both groups have similar high total effective rates (Table 2).

3.3.2 Comparison of the NRS score

After treatment, the NRS scores of both groups decreased significantly, and the intra-group differences were statistically significant ($P<0.01$). There was no significant difference in the NRS score between the two groups ($P>0.05$), indicating that both groups can reduce low back pain with an identical effect (Table 3).

3.3.3 Comparison of the BROM

After treatment, the BROM of both groups increased significantly, and the intra-group differences were statistically significant ($P<0.01$). The BROM in the directions of extension and left lateral flexion of the TCM points group was higher than that of the TUNG's extra points group, and the difference was statistically significant ($P<0.01$, $P<0.05$) as shown in Table 4 and Table 5.

3.3.4 Comparison of the back strength

After treatment, there was no significant difference found in the back strength in the intra-group and the between-group comparisons ($P>0.05$), indicating that neither group could foster back strength in patients with CLBP (Table 6).

**Table 2 Comparison of the efficacy between the two groups** **Unit: case**

| Group | *n* | Markedly effective | Effective | Invalid | Total effective rate (%) |
|---|---|---|---|---|---|
| TUNG's extra points | 28 | 25 | 2 | 1 | 96.4 |
| TCM points | 30 | 25 | 4 | 1 | 96.7 |

Note: TCM=Traditional Chinese medicine.

**Table 3 Comparison of the NRS score between the two groups ($\bar{x} \pm s$)** **Unit: point**

| Group | *n* | Before treatment | After treatment | *t*-value | *P*-value |
|---|---|---|---|---|---|
| TUNG's extra points | 28 | 7.75±2.38 | 1.50±1.43 | 13.229 | 0.001 |
| TCM points | 30 | 7.27±2.50 | 1.45±1.42 | 12.606 | 0.001 |
| *t*-value | | 0.752 | 0.134 | | |
| *P*-value | | 0.483 | 0.894 | | |

Note: NRS=Numeric rating scale; TCM=Traditional Chinese medicine.

| © The author(s) for open access article 2023

**Table 4 Comparison of the BROM of flexion and extension between the two groups ($\bar{x} \pm s$) Unit: °**

| Group | *n* | Flexion | | | | Extension | | | |
|---|---|---|---|---|---|---|---|---|---|
| | | Before treatment | After treatment | *t*-value | *P*-value | Before treatment | After treatment | *t*-value | *P*-value |
| TUNG's extra points | 28 | 84.95±25.39 | 102.20±18.31 | -3.659 | 0.001 | 16.99±8.31 | 22.15±7.60 | -3.301 | 0.003 |
| TCM points | 30 | 89.56±18.38 | 110.32±14.75 | -6.188 | 0.001 | 19.55±9.06 | 28.37±7.91 | -4.829 | 0.001 |
| *t*-value | | -0.797 | -1.864 | | | -1.116 | -3.055 | | |
| *P*-value | | 0.429 | 0.068 | | | 0.269 | 0.003 1) | | |

Note: BROM=Back range of motion; TCM=Traditional Chinese medicine.

**Table 5 Comparison of the BROM of left lateral flexion and right lateral flexion between the two groups ($\bar{x} \pm s$) Unit: °**

| Group | *n* | Left lateral flexion | | | | Right lateral flexion | | | |
|---|---|---|---|---|---|---|---|---|---|
| | | Before treatment | After treatment | *t*-value | *P*-value | Before treatment | After treatment | *t*-value | *P*-value |
| TUNG's extra points | 28 | 27.38±9.11 | 33.80±7.94 | -4.479 | 0.001 | 28.79±9.24 | 37.39±9.34 | -5.173 | 0.003 |
| TCM points | 30 | 31.21±8.10 | 38.78±9.51 | -4.441 | 0.001 | 29.93±8.70 | 41.14±10.39 | -5.912 | 0.001 |
| *t*-value | | -1.694 | -2.155 | | | -0.484 | -1.444 | | |
| *P*-value | | 0.096 | 0.035 | | | 0.630 | 0.154 | | |

Note: BROM=Back range of motion; TCM=Traditional Chinese medicine.

**Table 6 Comparison of the back strength between the two groups ($\bar{x} \pm s$) Unit: kg**

| Group | *n* | Before treatment | After treatment | *t*-value | *P*-value |
|---|---|---|---|---|---|
| TUNG's extra points | 28 | 40.18±15.15 | 42.14±13.52 | -0.999 | 0.327 |
| TCM points | 30 | 37.27±16.10 | 39.48±12.84 | -0.958 | 0.346 |
| *t*-value | | 0.708 | 0.768 | | |
| *P*-value | | 0.482 | 0.445 | | |

Note: TCM=Traditional Chinese medicine.

## 4 Discussion

According to the study results, the general data of the volunteers in both groups were not significantly different, so there was no effect from confounding variables in this study. Regarding the NRS score, the severe low back pain of the volunteers in both groups before treatment decreased to mild pain after treatment. In the between-group NRS score comparison, the two acupuncture methods were not significantly different in reducing low back pain.

The effectiveness of the acupuncture at the TUNG's extra points in reducing low back pain has been confirmed and complied with TUNG's acupuncture in the explanation that Dabai (22.04) is reflexive to the lungs and upper organs, and Linggu (22.05) is reflexive to the back and lower organs[23]. Therefore, both points can treat CLBP and stimulate the meridian flow in the upper and lower parts of the body. Moreover, Linggu (22.05) and Dabai (22.04) are the points on the Large Intestine Meridian (5:00-7:00), which is closely related to the Kidney Meridian (17:00-19:00) according to Chinese clock opposites. As the Kidney Meridian has a close relationship with the lumber area, both points mutually nourish the kidney while curing the low back pain effectively[29]. This is consistent with the study of NIU C, *et al*[30] regarding acupuncture at TUNG's extra points on both hands at Linggu (22.05) and Dabai (22.04) with 160 acute low back pain patients. Their study found that one session of acupuncture at the TUNG's extra points could immediately reduce low back pain. Meanwhile, the effectiveness in reducing low back pain by acupuncturing at the TCM points conformed to the gate control theory by puncturing the needles to stimulate nerve fibers to stop sending pain signals to the brain[31]. This is consistent with the study of LOIZIDIS T, *et al*[32] on acupuncture to reduce CLBP by puncturing the needles at the painful area at $L_2$-$L_5$ Jiaji (EX-B2) points of the lumber spine and Ashi points (trigger points). They found that acupuncture in the painful areas and puncturing all the muscle groups could improve pain relief and functional balance of the low back.

However, the between-group NRS score comparison results in this study differ from the study of XU X B[33], which reported that the acupuncture at the TUNG's extra points could reduce low back pain better than acupuncture at the TCM points. This difference might be due to the larger sample size of XU's study with 92 volunteers in the group getting the acupuncture at the

TUNG's extra points and 46 volunteers in the group getting the acupuncture at the TCM points within a treatment period of two weeks. In contrast, the present study had fewer volunteers and a shorter treatment period, so these factors may cause the between-group NRS score comparison results not to be significantly different.

Regarding the within-group BROM, the group getting the acupuncture at the TUNG's extra points showed an increase in BROM in all four directions, similarly to the group getting the acupuncture at the TCM points. In the between-group BROM comparison, however, the increase in BROM in the directions of extension and left lateral flexion in the group getting acupuncture at the TUNG's extra points was significantly lesser than in the group getting acupuncture at the TCM points. At the same time, acupuncture at the TCM points could increase BROM in all directions. These findings are consistent with the study of WANG X, *et al*[34], which reported that acupuncture at the TCM points could increase BROM in all directions. In addition, these findings are in accordance with the traditional acupuncture theory that the acupuncture mechanism is helpful in expanding the blood vessels at the punctured points[15], making the capillaries expand to nourish the body tissues, circulating blood, and relaxing muscles at the punctured area, so that the body movement is better[35]. However, the acupuncture at the TUNG's extra points is performed at the distal points and not at the direct back muscle area. Therefore, the back muscles do not relax, according to this theory. Although the pain NRS score reduced to the level at which the low back can move better than it did before treatment, some back muscles still remained stiff and convulsive, and some back parts could not move in some directions.

In terms of the low back strength, it did not show significant changes in either of the two groups after treatment. Moreover, the between-group comparison showed that the low back strength was not significantly different between the two groups. This might be due to the avoidance of touch during the outbreak of COVID, which might have obstructed the volunteers' posture arrangement. Some volunteers might be tested improperly, so that the scores might get some errors[36].

According to the results of this study, the acupuncture at the TUNG's extra points is equivalent to the acupuncture at the TCM points in the dimensions of NRS score and BROM in the directions of flexion and right lateral flexion. Therefore, the acupuncture at the TUNG's extra points can be used for treating elderly patients with CLBP in rural areas, who are usually cramped and lack the necessary facilities for reducing pain and increasing BROM. Moreover, acupuncture at the TUNG's extra points can be used for patients who cannot receive the treatment at the TCM points due to injuries and other pathological conditions in the back area, an inconvenience in making a prone position or in taking off their shirts/blouses to show the back area, a fear of acupuncture. Therefore, this acupuncture method can be an alternative method for treating elderly patients and patients with such limitations. However, this study lacks follow-up for long-term treatment results. In addition, the number of volunteers should be increased to make the between-group difference clearer. Therefore, future studies are required.

To conclude, acupuncture at the TUNG's extra points can reduce CLBP and increase BROM in flexion and right lateral flexion equivalently to acupuncture at the TCM points, particularly for elderly patients with CLBP living in rural area. Therefore, acupuncture at the TUNG's extra points for elderly patients with CLBP should be supported in Thailand.

**Conflict of Interest**

The authors declare that there is no potential conflict of interest in this article.

**Acknowledgments**

This work was supported by the Human Research Ethics from the University of Phayao (No. 1.3/029/64) and was registered in the Clinical Trials Registry (No. TCTR20220306001).

**Statement of Informed Consent**

Informed consent was obtained from all individual participants.

Received: 16 March 2022/Accepted: 27 June 2022
